# On the Characterization of Intermediates in the Isodesmic Aggregation Pathway of Hen Lysozyme at Alkaline pH

**DOI:** 10.1371/journal.pone.0087256

**Published:** 2014-01-28

**Authors:** Vijay Kumar Ravi, Tulsi Swain, Nividh Chandra, Rajaram Swaminathan

**Affiliations:** Department of Biotechnology, Indian Institute of Technology Guwahati, Guwahati, Assam, India; Aligarh Muslim University, India

## Abstract

Protein aggregation leading to formation of amyloid fibrils is a symptom of several diseases like Alzheimer’s, type 2 diabetes and so on. Elucidating the poorly understood mechanism of such phenomena entails the difficult task of characterizing the species involved at each of the multiple steps in the aggregation pathway. It was previously shown by us that spontaneous aggregation of hen-eggwhite lysozyme (HEWL) at room temperature in pH 12.2 is a good model to study aggregation. Here in this paper we investigate the growth kinetics, structure, function and dynamics of multiple intermediate species populating the aggregation pathway of HEWL at pH 12.2. The different intermediates were isolated by varying the HEWL monomer concentration in the 300 nM—0.12 mM range. The intermediates were characterized using techniques like steady-state and nanosecond time-resolved fluorescence, atomic force microscopy and dynamic light scattering. Growth kinetics of non-fibrillar HEWL aggregates were fitted to the von Bertalanffy equation to yield a HEWL concentration independent rate constant (*k* = (6.6±0.6)×10^−5^ s^−1^). Our results reveal stepwise changes in size, molecular packing and enzymatic activity among growing HEWL aggregates consistent with an isodesmic aggregation model. Formation of disulphide bonds that crosslink the monomers in the aggregate appear as a unique feature of this aggregation. AFM images of multiple amyloid fibrils emanating radially from amorphous aggregates directly confirmed that on-pathway fibril formation was feasible under isodesmic polymerization. The isolated HEWL aggregates are revealed as polycationic protein nanoparticles that are robust at neutral pH with ability to take up non-polar molecules like ANS.

## Introduction

Accumulation of ordered protein aggregates like amyloid fibrils in the intra or extracellular regions of human body is a common symptom among protein misfolding diseases [1]. For example, single amino acid mutations in human lysozyme (closely related to HEWL in amino acid sequence) have been reported to cause systemic non-neuropathic amyloidosis where in amyloid deposits of mutant lysozyme were found in kidneys, gastrointestinal tract, spleen and liver of affected patients [2]. For effective treatment of such diseases, it is important to understand the molecular mechanisms behind aggregation. Protein polymerization mechanisms are broadly classified under two prevalent models, namely the nucleation-elongation polymerization model and the isodesmic polymerization model [3], [4]. The latter however, is rarely encountered with few reports in protein literature till date [5], [6]. The absence of a critical concentration in isodesmic polymerization implies that aggregation can occur at nanomolar monomer concentrations which can be exploited to investigate molecular features of aggregates. Several questions that arise are: **A**) Is it possible to monitor the growth of aggregates at multiple monomer concentrations and extract their kinetic parameters? **B**) Can intermediates in isodesmic aggregation be isolated and characterized? **C**) Can amyloid fibrils originate under isodesmic aggregation in the dilute monomer concentration regime? **D**) Do initial amorphous aggregates formed under isodesmic mechanism convert into amyloid fibrils (on-pathway) or are fibrils formed by a separate pathway (off-pathway)? This work attempts to answer the above questions.

Hen eggwhite lysozyme (HEWL), is a well known enzyme that cleaves the glycosidic bond between alternating sugar residues of peptidoglycan in bacterial cell walls [7]. It is widely employed as a food preservative [8]. HEWL is an excellent model to investigate protein aggregation. It has been shown to form aggregates under a variety of conditions like acidic pH, alkaline pH, presence of zero disulphide bond, ethanol and guanidine hydrochloride [9]. The aggregation and amyloid formation among different proteins in the lysozyme family has been reviewed previously [10].

HEWL, whose pI is 11.3 [11], is polycationic at acidic and neutral pH. Consequently aggregation of polycationic HEWL at acidic pH must be forced by high temperatures and mechanical agitation [12]. In contrast, HEWL aggregation is facile and spontaneous with rise in pH as shown previously [13], [14]. Subsequent work from our lab has revealed that aggregation of HEWL at pH 12.2 is a slow (0—12 hours) but concentration dependent spontaneous process at 298 K [15]. This aggregation was instantly triggered by negligible net charge and increased exposure of hydrophobic surfaces in HEWL at high pH. Interestingly, we discovered that HEWL aggregates at pH 12.2 are further reinforced by formation of intermolecular disulfide bonds after ∼100 hours [16].

In this paper, we attempt to unravel the mechanism of HEWL aggregation at pH 12.2 by characterizing the molecular features of different aggregate species populated at different times under different HEWL monomer concentrations.

## Materials and Methods

### Protein labeling

HEWL was covalently labeled with dansyl chloride (2-dimethyl aminonaphthalene-6-sulfonyl chloride) following the protocol recommended by Molecular Probes with minor modification as reported previously[15]. For labeling with dabcyl (4-((4-(dimethylamino) phenyl) azo) benzoic acid succinimidyl ester), the protocol suggested by Molecular Probes was followed. The HEWL, dansyl and dabcyl concentrations were estimated by measuring the absorbance at 280 nm (ε  =  37,970 M^−1^ cm^−1^ [17]), 380 nm (16,000 M^−1^cm^−1^) and 453 nm (32,000 M^−1^cm^−1^), respectively. The protein to dye labeling ratio in the conjugate were consistently between 2—3. Henceforth, 0.5 µM dansyl (or dabcyl)-conjugated HEWL refers to dye concentration. If the protein/dye labeling ratio was 2.0, this would contain 1.0 µM of HEWL of which 50% of HEWL is likely to be unlabeled.

### Sample preparation and incubation

HEWL stock (10 mg/mL or 699 µM) was freshly prepared before use in deionised water (MilliQ, Millipore, India). For control experiments this stock was diluted in 50 mM, pH 7 phosphate buffer. For inducing aggregation, the stock was diluted in 50 mM, pH 12.2 phosphate buffer. In between measurements, all samples were incubated at 22–28°C. All HEWL concentrations refer to monomeric HEWL. All measurements were performed at 25°C.

### Steady-state fluorescence measurements

All steady-state fluorescence measurements were performed using Fluoromax-3 spectrofluorometer (Jobin-Yvon Horiba Inc., USA). To minimize photobleaching, excitation light shutter was kept open only when recording fluorescence.

*Intermolecular Förster resonance energy transfer* (FRET) measurements for HEWL monomer concentrations 3—120 µM, were carried out by mixing 0.5 µM dansyl-conjugated HEWL (donor) and 0.5 µM dabcyl-conjugated HEWL (acceptor) with excess of unlabeled HEWL. Thus, unlabeled HEWL was at most 2 µM for 3 µM HEWL sample, while it was similarly 119 µM for 120 µM HEWL sample. The 0.3 µM HEWL sample was prepared by diluting the 3 µM HEWL sample above, by 10-fold in the appropriate buffer. Samples were excited at 380 nm (slit width 1 nm) and emission collected between 400 and 700 nm (slit 5 nm). For 0.3 µM samples, slit width was kept 10 nm. FRET efficiency was calculated from average integrated total emission counts under emission curve of donor alone (F_D_) & donor-acceptor mixture (F_DA_) after subtraction of blank using equation 1.
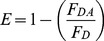
(1)


For *ANS* (8-Anilino-1-naphthalene sulfonic acid ammonium salt) *binding experiments*, the stock concentration of ANS (1 mg/mL equivalent to 3.2 mM) in water was verified using extinction coefficient of 4,950 M^−1^cm^−1^ at 350 nm. The HEWL concentration dependent ANS binding assay was performed by employing a constant HEWL (3 µM) and constant ANS (10 µM) concentration in cuvette for pH 7 (3 µM) or 12.2 (3 to 120 µM) incubated samples. For 0.3 µM HEWL sample (in pH 12.2), protein was retained at 0.3 µM, while ANS was added to a final concentration of 10 µM in cuvette. Steady state fluorescence intensity was measured after excitation at 380 nm (slit  =  1 nm). Emission (slit  =  16 nm for 0.3 µM, 8 nm for rest) was collected in the range 400-600 nm as reported previously [15]. For experiments related to binding of ANS to HEWL aggregates transferred to pH 7, 10 µM ANS was employed in all samples, while identical slit widths were maintained throughout. Integrated fluorescence intensity was computed by calculating area under emission spectrum after subtraction of blank.

The fluorescence of tryptophan was quenched by adding KI (0 to 0.8 M) as described earlier [18]. Fluorescence quenching studies were performed employing a dilute concentration of HEWL (1.2 µM) in cuvette for 120 µM and 3 µM HEWL samples to avoid inner filter effect. For 0.3 µM HEWL sample, protein was retained at 0.3 µM, while KI was added to the desired concentration in cuvette. NATA (5 µM in pH 12.2 buffer) was used as a control. Steady state fluorescence of tryptophan in HEWL was measured by exciting at 295 nm (1 nm slit) and collecting emission between 310–400 nm (slit 20 nm for 0.3 µM, 10 nm for rest). The slopes reported from Stern-Volmer plots were averaged from three sets of experiments. See below for tryptophan lifetime measurement.

### Steady-state fluorescence anisotropy measurements

The steady-state fluorescence anisotropy, r_ss_ was measured after G-factor correction and dark counts subtraction as described previously [15], [16]. For HEWL monomer concentrations 3—120 µM, the concentration of dansyl-conjugated HEWL was 0.6 µM, while remaining protein was unlabeled. Similarly 0.3 µM HEWL contained 0.16 µM of dansyl-conjugated HEWL. For experiments related to robustness of polycationic HEWL aggregates transferred to pH 7, the 120 µM and 40 µM HEWL samples each contained 1 µM dansyl-conjugated HEWL before dilution.

Dansyl-labeled HEWL was excited at 380 nm (slit width  =  1 nm) and emission at 438 nm was collected with a slit width between 5—10 nm. Each measurement was done in duplicate, while data reported are averages of three such measurements. The increase in r_ss_ with time, in pH 12.2, for different HEWL concentrations was modeled by the von Bertalanffy equation below:


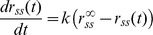
, which can be solved to yield
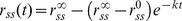
(2)


Here, refers 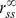
to r_ss_ at t  =  infinity, while 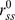 refers same at *t*  =  0. *k* denotes the rate constant for rise in anisotropy which corresponds to growth of HEWL aggregates.

### Time-resolved fluorescence measurements

Time-resolved fluorescence intensity and anisotropy (G-factor corrected) measurements were carried out in LIFE SPEC II spectrometer (Edinburgh Instruments, Livingston, UK) operating in TCSPC mode, collecting emission decay in 4096 channels using a microchannel plate PMT. Mean fluorescence lifetime of tryptophan was measured by exciting samples with 295 nm nanosecond pulses (Instrument Response Function (IRF) fwhm was ∼0.6 ns, Nano LED) and collecting subsequent emission at 350 nm with a temporal resolution of 4.883 ps/channel. HEWL-dansyl conjugates were excited with 375 nm (EPL-375, picosecond pulsed diode laser, IRF fwhm ∼150 ps) and emitted fluorescence from dansyl probe was collected at 440 nm with a temporal resolution of 24.414 ps/channel. For tryptophan lifetime measurements, HEWL samples (3—120 µM) were diluted to 1.2 µM in the cuvette, while 0.3 µM samples were used as is. For dansyl probe lifetime measurements, the concentration of dansyl-conjugated HEWL was kept at 1.0 µM in HEWL samples (3—120 µM), while rest of the protein was unlabeled. Similarly 0.3 µM contained 0.1 µM of dansyl-conjugated HEWL. Each data reported is the average of three measurements. Intensity decays were analysed by iterative reconvolution using the Marquardt-Levenberg algorithm to extract lifetimes (τ_i_) and amplitudes (α_i_) as given in equation below.


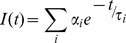
 where *i*  =  1—3 and 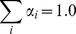 (3)

Mean fluorescence lifetime, 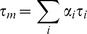 (4)

The raw (G-factor corrected) anisotropy decays were tail-fitted using a sum of two exponentials (equation 5), yielding two rotational correlation times. 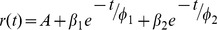
(5)


Here, A is a constant dependent on G-factor, β_i_ denotes the amplitude for φ_i_, φ_1_ and φ_2_ refer to the fast and slow rotational correlation times, respectively. The slower rotational correlation time (φ_2_) corresponds to global rotational motion of the whole HEWL aggregate. As the 0.15 ns IRF pulse-width is negligibly small in comparison to the time scale of protein rotational motion (> 4 ns), the extracted values of φ_2_ by this tail-fit approach are not affected by consequences of IRF convolution. It must be emphasized that φ_2_ values reported here for higher monomer concentrations of HEWL are more *reliable* compared to our earlier reports for 40 [15] and 120 [16] µM for two reasons: a) The anisotropy decay here is sampled over a larger time window (0—100 ns), yielding a *complete decay* for analysis, b) The decay here is acquired in 4096 time channels with a time resolution of 24.4 ps/channel compared to 1024 channels with 113 ps/channel earlier.

### Multiangle and Dynamic light scattering

Multiangle light scattering measurements were performed on WYATT DAWN 8 multi-angle light scattering system which has 8 detectors (1 for dynamic and remaining for static light scattering) and uses a 658 nm laser. For molecular weight measurements seven day old 120 µM HEWL samples in pH 12.2 were diluted 4.3 fold in 20 mM Tris buffer (pH 7) containing 50 mM NaCl and filtered through 0.2 µm filter.

Dynamic light scattering experiments were carried out using the Spectroscatterer 201 (RiNA GmbH, Germany) with a He-Ne laser providing light of 690 nm wavelength and an output power in the range of 10–50 mW. HEWL samples (120 µM, 30 µL) were placed in a quartz cuvette and measured at a constant temperature of 293 K using an autopilot function accumulating 30 measurements per sample. The corresponding molecular sizes were calculated by standard procedures.

### Lysozyme activity measurements

The enzymatic activity of HEWL was measured as described earlier [16], [19]. Briefly, a suspension of *Micrococcus lysodeikticus* (78 µg/mL) in assay buffer (50 mM phosphate, pH 7.0), was mixed with HEWL, diluted to 70 nM from aggregate/pH 7 sample. Activity assays could not be done with 0.3 µM monomer concentration as lower dilutions (<10 fold) are likely to affect the final pH. Each assay was performed in triplicate and averaged over multiple sets of experiments.

### Imaging aggregates by Atomic Force Microscopy

HEWL samples (10 µl) were applied on to freshly cleaved mica in the presence of 10 mM Mg^2+^. After a few minutes, these were rinsed with 0.2 µm filtered deionized water to remove unadsorbed sample and were dried under nitrogen. Samples were imaged in air under AAC or MAC MODE (non contact) in PICO PLUS^TM^ AFM purchased from Molecular Imaging, USA. Cantilever type PPP-MFMR-20 (resonance frequency, 60—70 kHz ;Nanosensors) was used for MAC mode, while type PPP-NCL-50 (resonance frequency, 150 kHz ; Molecular Imaging) was used for AAC mode. Images were acquired digitally at a scan speed of 1—2 lines/second with 256 data points per line.AFM images were acquired at least three times for every sample condition.

### Materials

Sodium dihydrogen phosphate dihydrate, potassium iodide, sodium bicarbonate and sodium thiosulphate of analytical grade, along with solvents like DMSO, DMF were purchased from Merck Limited, Mumbai. ANS (8-Anilino-1-naphthalene sulfonic acid ammonium salt), NATA (N-Acetyl-L-tryptophanamide), DTNP (2,2′-dithiobis(5-nitropyridine)),lyophilized cells of *Micrococcus Lysodeikticus* (ATCC 4698), hen eggwhite lysozyme (HEWL, L6876/L7651) were procured from Sigma-Aldrich Chemicals Pvt. Ltd., India. Dansyl chloride and Dabcyl SE were purchased from Invitrogen, USA. AFM Cantilevers PPP-NCL-50 (Point probe Plus/ Non- contact/ Long cantilever, part 65-262P) was purchased from Molecular Imaging, USA. Muscovite mica, V-1 quality was purchased from Electron Microscopy Sciences, USA.

## Results

Our previous work on HEWL aggregation at pH 12.2 had shown that progress of HEWL aggregation was dependent on monomer concentration (henceforth referred as **[**M**])** in the range of 4—200 µM [15]. However, it was not clear how change in [M] affects growth kinetics, internal features, enzymatic activity, size and other characteristics of the aggregates as a function of time. Hence our objective was to track the progress of HEWL aggregation for [M] between 0.3—120 µM, using multiple analytical tools to unravel the characteristics of aggregates as they grow with time. [M] > 120 µM was avoided to keep all aggregates in soluble form.

### HEWL aggregation observed using intermolecular FRET

**Figure 1:**
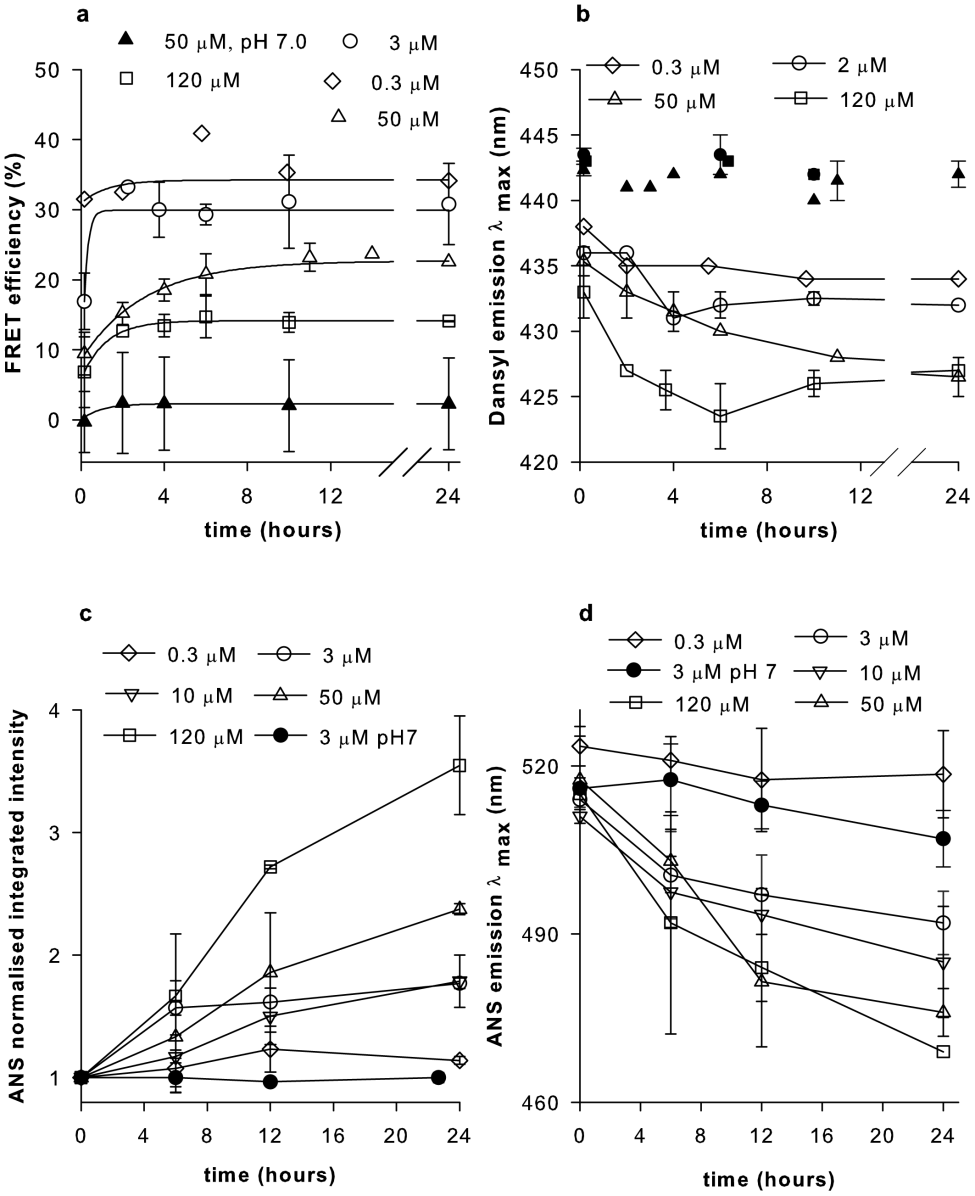
Association of monomers, changes in polarity inside the aggregates. **a,** The efficiency of fluorescence energy transfer between 0.5 µM dansyl-labeled HEWL (donor) mixed with 0.5 µM dabcyl-labeled HEWL (acceptor) in presence of excess unlabeled HEWL at different time intervals post-incubation for 3, 50 & 120 µM HEWL in pH 12.2 and 50 µM in pH 7.0 are shown. For 0.3 µM, a 10 fold dilution of 3 µM sample was employed. The data were fitted by an equation identical to Eq. 2 after replacing anisotropy with FRET efficiency. **b,** Changes in emission λ_max_ of dansyl-labeled HEWL (alone) as above. **c**, Changes in emission λ_max_, and **d**, Integrated normalized emission intensity of ANS (10 µM) in presence of HEWL are shown for different time intervals post-incubation of HEWL at concentrations 0.3, 3, 10, 50, & 120 µM in pH 12.2 and 3 µM in pH 7. For all data in panel, filled symbols indicate pH 7, while unfilled symbols represent pH 12.2. Each HEWL concentration is represented by a unique symbol. Error bars, SD; N  =  3.

The association of HEWL monomers is the first step in aggregation, as revealed by the rise in intermolecular Förster resonance energy transfer (FRET) efficiency [20] with time when donor and acceptor labeled HEWL were mixed with excess of unlabeled HEWL at pH 12.2 (**Figure 1a**). The apparent faster kinetics and higher magnitude of FRET efficiency at lower (3 and 0.3 µM) as compared to higher (50 and 120 µM) [M] may be attributed to the effect of dilution. There are 4 unlabeled proteins for every labeled HEWL molecule at 3 and 0.3 µM, compared to 238 at 120 µM. The prospect of having more acceptors per donor in an oligomer is also higher at lower concentrations. The marginal increase in FRET efficiency in 0.3 µM hints at relatively smaller size (more proximity) of oligomers at this concentration compared to 3 µM. HEWL can exist as weak dimer at pH 7 as shown previously [13], [21].

### Internal milieu of HEWL aggregates

Next, we look at the molecular interior of HEWL complexes at different times as they grow using polarity sensitive fluorescent probes. The dansyl probe fluorescence emission intensity increases, while its wavelength decreases as surroundings become more non-polar [22]. **Figure 1b**, reveals a developing hydrophobic environment around the dansyl probe for 120 µM HEWL, while lower concentrations display progressively less non-polarity. A similar concentration dependent increase in intensity with time was also observed. Moreover, fluorescence quenching of six tryptophans in HEWL by iodide revealed reduced solvent exposure of tryptophans with increasing [M] at pH 12.2 (**Figure S1**).

A covalently conjugated probe like dansyl and an intrinsic probe like tryptophan, are constrained in exploring the available microenvironments, hence a freely diffusing probe like ANS was tried. ANS preferentially and non-covalently binds to exposed hydrophobic regions of protein displaying enhanced fluorescence intensity and blue-shifted emission compared to water [23]. In **Figure 1c**, the increase in normalized total intensity with time is best highlighted by 120 µM, followed below by decreasing [M]. **Figure 1d** shows an opposite trend in emission λ_max_ of ANS with time.ANS is insensitive at 0.3 µM due to low protein concentration, while at pH 7, the unavailability of exposed hydrophobic sites in the native protein may make ANS unresponsive.

The concentration dependent trends in tryptophan shielding, emission spectra of ANS and dansyl probes with time highlight a quantitative increase in accessible hydrophobic pockets inside growing HEWL aggregates with increasing [M]. Growing HEWL aggregates may acquire non-polar interiors when monomers cluster and later undergo increased molecular packing. As a consequence, HEWL aggregate volume must increase when [M] is raised.

### Enzymatic activity in HEWL aggregates

**Figure 2:**
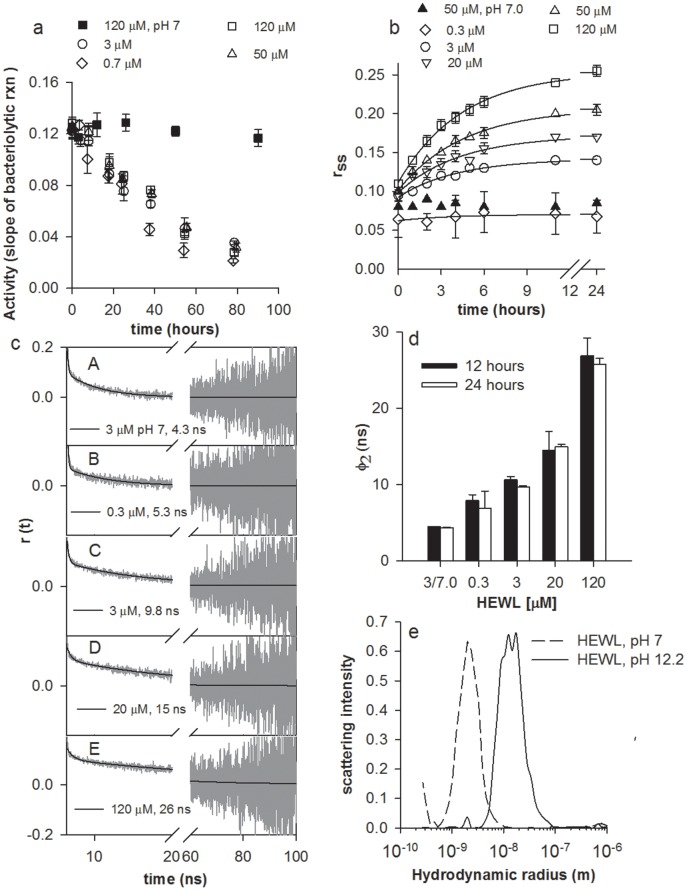
Activity and size of HEWL aggregates. **a,** Bacteriolytic activity (in ▵Abs/min) of HEWL (diluted to 70 nM in assay cuvette) measured at pH 7 with pppppppwith *M. lysodeikticus* is highlighted at different time intervals subsequent to incubation of HEWL at concentrations 0.7, 3, 50 & 120 µM in pH 12.2 and 120 µM in pH 7. **b**, Changes in steady state fluorescence anisotropy (r_ss_) of dansyl-HEWL conjugates at different time intervals in presence of 0.3, 3, 20, 50 & 120 µM of HEWL incubated in pH 12.2 and 50 µM in pH 7 is shown along with fitted curve for pH 12.2 (Eq. 2, Table 1). **c**, Nanosecond time-resolved fluorescence anisotropy decay of dansyl-HEWL conjugates after 24 hours for following protein concentrations (pH): A: 3 µM (7); B—E: 0.3, 3, 20 and 120 µM (12.2). The dark continuous line depicts the fit and extracted value for φ_2_ using tail fit analysis (Table S1). **d**, Average global rotational time (φ_2_) for 0.3—120 µM HEWL at pH 12.2 and 3 µM at pH 7 (extreme left) after 12 & 24 hours (Table S2). **e**, Size of HEWL aggregates revealed by dynamic light scattering after incubation of 120 µM HEWL in pH 12.2 (continuous line) and pH 7 (dashed line) for 26 hours. Peaks at R_h_ ∼2.0 nm indicate HEWL monomer, while those at 12.5 and 17.4 nm along with shoulders at R_h_ ∼9.0 and 28.7 nm reveal multimeric aggregates. Error bars, SD; N  =  3.

HEWL enzymatic activity provides a tool to quantify the population of native functional protein that can be reversibly recovered from the protein in non-native state. **Figure 2a** shows the slow loss of HEWL activity in pH 12.2, which reflects steady depletion of active HEWL with time owing to increasingly irreversible unfolding. Intriguingly, the absolute activity, at any given time, remains fairly invariant across different [M], indicating: a) loss of activity due to unfolding is concentration independent; b) aggregates possess similar enzymatic activity irrespective of [M]. It was previously shown that after initial 60 minutes, aggregates formed with 40 µM HEWL, do not dissociate on exposure to neutral pH [24]. These results reveal that aggregates transferred into neutral pH at early times (0—40 hrs) retain significant activity, but gradually fail to do so later as they grow up.

### Size of growing HEWL aggregates

To track molecular volume of growing HEWL aggregates, the Brownian rotational motion of the whole aggregate was measured in alkaline pH as a function of [M] and time using the fluorescence anisotropy [25] of covalently conjugated dansyl probe. The long fluorescence lifetime (∼13 ns) of the dansyl probe is ideal to monitor the slow global rotation of large aggregates over a time window of 0—100 ns. **Figure 2b**, shows that magnitude of increase in r_ss_ is strongly concentration dependent, revealing the highest increase for 120 µM, followed by decreasing [M]. The fluorescence lifetime of the same dansyl-HEWL aggregates were nearly constant under similar conditions (**Figure S2**) highlighting that rise in r_ss_ solely reflects growth-dependent increase in molecular volume of aggregate that slows down the rotational motion. A clear absence of lag phase in the growth of HEWL aggregates is observed consistent with FRET data **(** **Fig. 1a** **)**. The growth of aggregates in Figure 2b was fitted to Equation 2 which is a model for restrictive growth [26]. This relation predicts that small oligomeric aggregates shall experience rapid growth in the beginning (when r_ss_(t) << 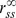
_)_, but at later times this growth slows down as r_ss_(t) approaches more close to maximum value (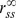). **Table 1** lists the fitted parameters. The fits reveal a fairly constant value for von Bertalanffy constant *k* across multiple [M]. This implies that rate constant for growth of aggregates is independent of monomer concentration. Overall, aggregation of HEWL follows a restrictive growth mechanism, where final size of aggregate (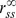), is limited by availability of free monomers.

**Table 1:** Parameters extracted (using eq. 2) from fits for growth in steady state anisotropy (r_ss_) shown in Figure 2b.

[HEWL]	pH	r_ss_^∞^	r_ss_^0^	*k* (hour^−1^)	R^2^
0.3 µM	12.2	0.070	0.062	0.274	0.46
3 µM	12.2	0.142	0.093	0.240	0.98
20 µM	12.2	0.172	0.098	0.231	0.97
50 µM	12.2	0.206	0.102	0.218	1.0
120 µM	12.2	0.254	0.117	0.224	1.0

To further confirm these results, time-resolved fluorescence anisotropy measurements were performed. **Figure 2c** shows the raw (G-factor corrected) anisotropy decay curves for HEWL-dansyl conjugates, after 24 hours of incubation at alkaline pH, at multiple concentrations. The slow rotational correlation time (φ_2_) is directly proportional to spherical hydrodynamic volume of HEWL aggregate as per the Stoke-Einstein equation. **Figure 2d** displays a summary of observations after 12 and 24 hours (see values in **Table S2**). At pH 7, φ_2_ was 4.4 ns, which is in good agreement with previous results [16], [27], corroborating a predominantly monomeric HEWL. At pH 12.2, we observe a progressive increase in φ_2_ as [M] increases from 0.3 µM to 120 µM. A spherical hydrated protein of molecular mass 12.1 and 71.3 kDa is calculated to yield φ_2_ of 4.4 and 26 ns, respectively [20]. Clearly, average volume of growing HEWL aggregate increases as [M] is raised.

Dynamic light scattering (DLS) experiments performed with unlabeled 120 µM HEWL incubated for 26 hours in pH 12.2 (**Figure 2e**) reveal a broad heterogeneous size distribution of aggregates, clearly arguing against selective increase in population of a specific size. Multi-angle light scattering (MALS) experiments revealed a molecular mass (2.3±0.4)×10^6^ Da with mean R_h_ near 24 nm (from DLS detector) in week old 120 µM HEWL (**Figure S3**). Interestingly in both DLS results above, a relatively small monomer population is consistently observed. The monomer appears to exist in equilibrium with larger aggregates. Thus the mean R_h_ ∼24 nm is likely to be larger if area weighted contribution from the small monomer peak is neglected. A spherical shape must yield a radius of ∼9.6 nm for the above molecular mass assuming a partial specific volume of 0.73 cm^3^/g and 0.23 cm^3^/g for the protein hydration [20]. The discrepancy in sizes revealed by DLS, MALS and time-resolved anisotropy imply that shapes of the aggregates could be asymmetrical. An elongated, cylindrical shape (like a prolate ellipsoid) could account for faster rotational depolarization of dansyl probe about the long axis in anisotropy data and create a perception of a large spherical body in DLS.

### Aggregate morphology

**Figure 3:**
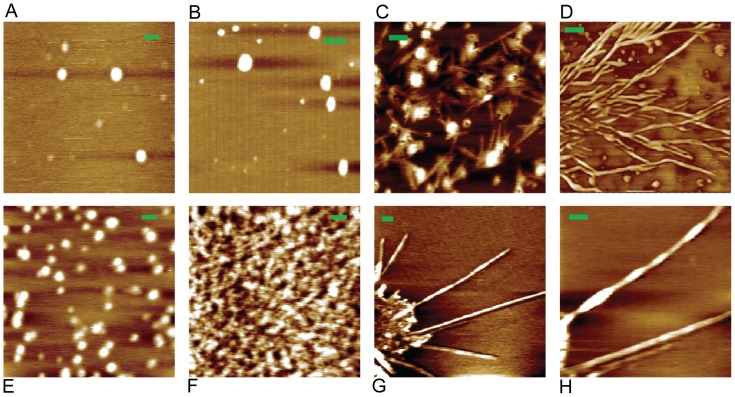
Morphology of HEWL aggregates. Observed morphology of HEWL aggregates after incubation in pH 12.2 (unless indicated otherwise) for 12 hours observed using non-contact mode atomic force microscopy with indicated monomer concentrations is shown. **A**: 3 µM; **B**: 120 µM; **C**: 0.3 µM; **D**: 120 µM; **E**: 0.3 µM; **F**: 3 µM in pH 7; **G**: 0.3 µM; **H**: fibril in ‘**G**’ rescanned at higher magnification; Scale bar  =  100 nm. Heights in Z-axis for all images were within 0—2 nm. See images of amyloid fibrils after 3 weeks in Figure S4.

AFM topology images (**Figure 3** A,B,E) show that amorphous hen lysozyme aggregates are well dispersed, essentially flat and its morphology noted as mostly spherical. A heterogeneous mix of sizes is visible. The aggregates clearly appear larger in size in comparison to monomer (Figure 3F). Amyloid fibrils with extensive branching, a rare event, were also observed as early as 12 hours after incubation at pH 12.2 (image D). Branching among HEWL amyloid fibrils has been observed by us previously too [28]. Interestingly, image C shows tiny straight and pointed fibril-like structures emanating from all amorphous aggregates. Image G reveals multiple fibrils originating from a single patch, while image H provides a magnified view of one newly formed amyloid fibril with the twist in the long axis clearly visible. Thus AFM images C, G and H in Figure 3 clearly show that growth of amyloid fibrils can be directly observed from amorphous aggregates in HEWL. **Figure S4** shows long, straight, tightly bunched amyloid fibrils observed at different [M] after 3—4 weeks of incubation at pH 12.2.

### Role of disulphide bonds

A uniformly slow decrease in –SH population was observed with aggregate growth for different HEWL monomer concentrations (**Figure S5**), hinting disulphide bond formation. Previous work has shown that HEWL aggregation at pH 12.2 is abolished when cysteine is maintained in a reduced form with DTT [16]. Additionally, blocking disulphide bond formation by modifying few but not all –SH groups using iodoacetamide produced drastically smaller HEWL aggregates in comparison to unblocked samples [29]. These results substantiate the vital role of intermolecular disulphide bridges in holding the aggregate together.

### Robustness of polycationic HEWL aggregates transferred to pH 7

**Figure 4:**
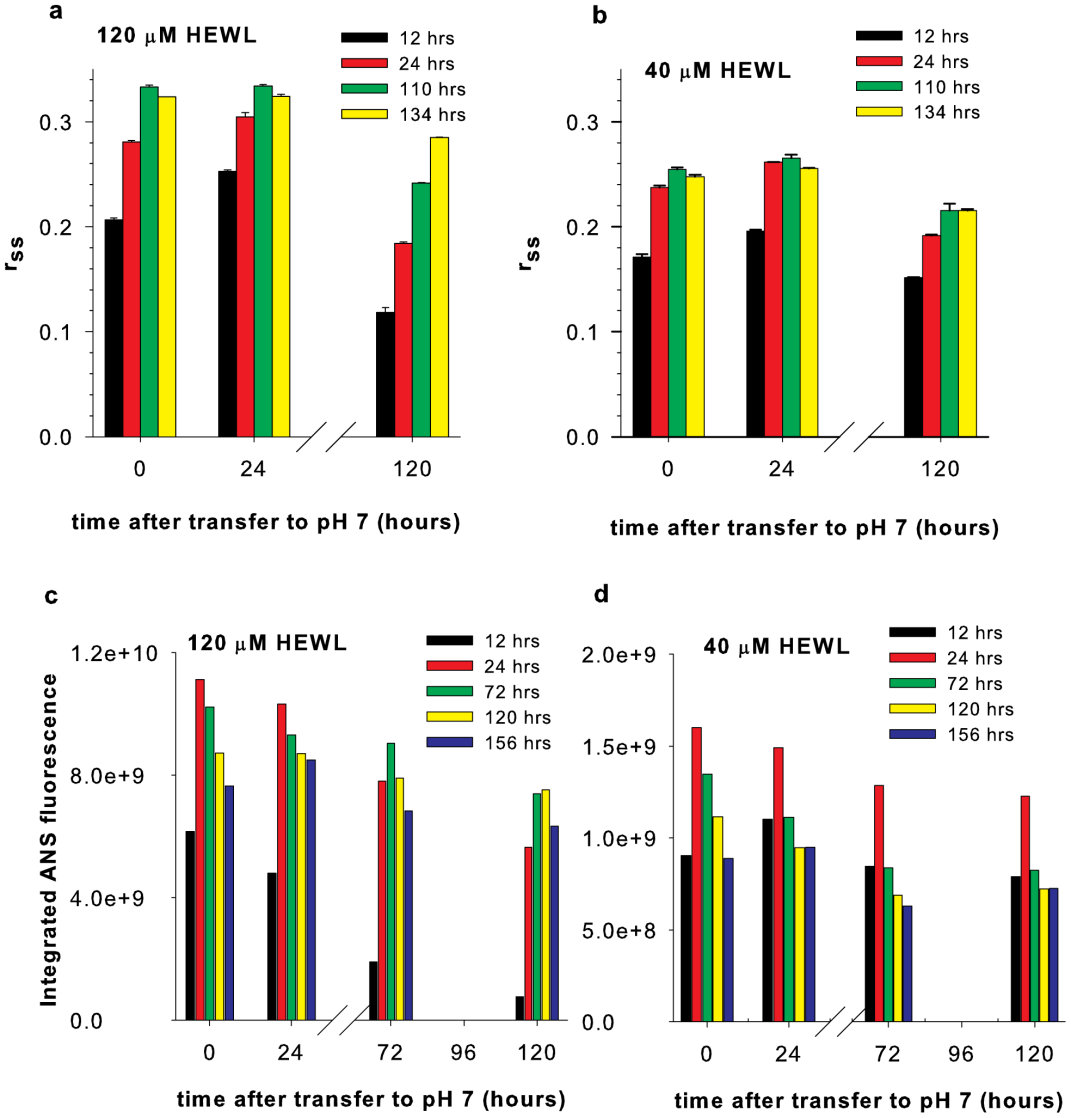
Stability of HEWL aggregates transferred to pH 7. **a,** Changes in dansyl r_ss_ after 10-fold dilution of 12, 24, 110 and 134 hours incubated 120 µM HEWL sample in pH 12.2 to 100 mM sodium phosphate buffer at pH 7 are shown. **b,** Changes in r_ss_ after a similar dilution with 40 µM HEWL sample is shown. **c,** Changes in total ANS fluorescence intensity after 10-fold dilution of 12, 24, 72, 120 and 156 hours incubated 120 µM HEWL sample in pH 12.2 into 100 mM sodium phosphate buffer at pH 7 are shown.**d,** Changes in total ANS fluorescence intensity after a similar dilution with 40 µM HEWL sample is shown. Error bars, SD; N  =  3.

**Figure 5:**
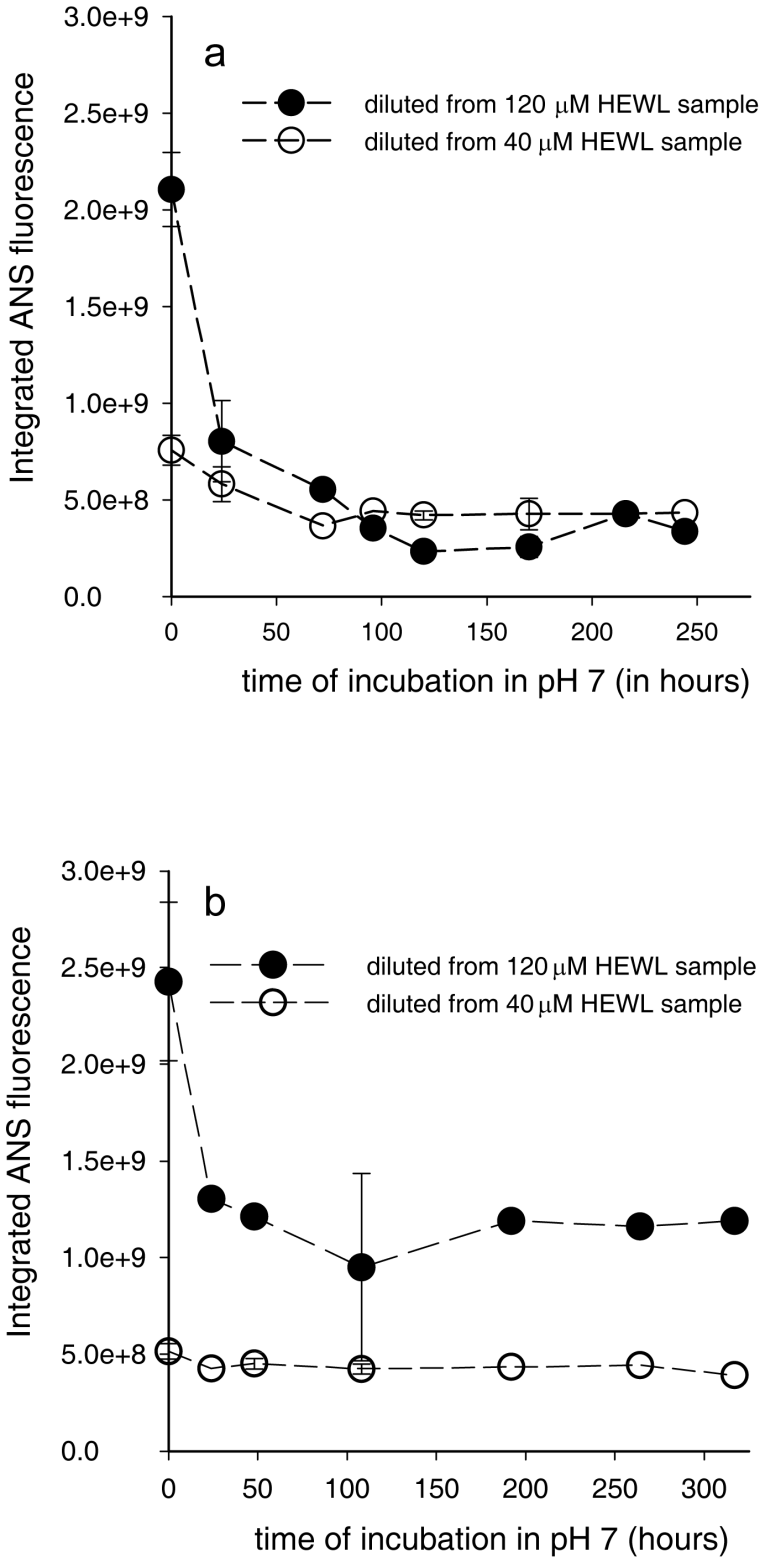
Binding of ANS to HEWL aggregates at equal concentration. Changes in total ANS fluorescence intensity with time after dilution of 144 (a) and 264 hours (b) incubated 120 µM (filled circles) and 40 µM (unfilled circles) HEWL samples in pH 12.2 into 100 mM sodium phosphate buffer at pH 7 are shown. To facilitate comparison, HEWL concentration interacting with ANS in cuvette was maintained constant at 4 µM in both (a) and (b). Error bars, SD; N  =  2.

An elegant way to ascertain the stability of HEWL aggregates formed in pH 12.2 is to test their intactness after transfer to neutral pH. Being polycationic at pH 7, coulombic repulsions can destabilize larger HEWL aggregates more than smaller. The robustness of aggregates was ascertained by: A) Observing the size and integrity of the dansyl-HEWL aggregates, from r_ss_ of dansyl probe, in a time-dependent way after transfer to pH 7, B) Measuring the ability of HEWL aggregates to take up a probe like ANS that displays enhanced fluorescence when bound to non-polar interior in a protein [30], [31]. **Figure 4a** shows a rise and dip in r_ss_ of transferred samples (during 0—120 hrs) when transferred at early times (12 and 24 hours of incubation) indicating disruption of aggregate later. However samples transferred at 110 and 134 hours significantly retain their r_ss_ values even after 5 days in pH 7 suggesting robust size and shape. **Figure 4b** shows that at 40 µM, the robustness is improved for HEWL aggregates transferred early also, perhaps due to less repulsions in a smaller aggregate. ANS binding data (**Figure 4c**) reveal that 12 hour aggregates are disintegrating quickly, while one day old samples acquiring shape with exposed interiors, take up ANS maximally, consistent with r_ss_ data above. Later samples, which are fairly well packed, reveal a steady and intense ability to bind ANS. A similar trend is observed with 40 µM HEWL also (**Figure 4d**), however here, like with r_ss_, ANS binds better in samples transferred early indicating better robustness. The 120 µM samples show significantly higher ANS fluorescence intensity and more blue-shifted spectra (**Figure S6**) compared to 40 µM. However, the ANS fluorescence intensity values in Figure 4c cannot be compared with Figure 4d as the HEWL concentrations interacting with ANS are different. **Figure 5** enables such comparison possible as HEWL concentration is maintained constant (4 µM) for both 120 and 40 µM samples. 120 µM HEWL samples that are transferred early (144 hours) show a tendency to lose ANS binding regions with time in pH 7 and approach ANS intensities that are comparable with 40 µM samples. In contrast, 120 µM HEWL samples that are transferred later (264 hours) retain higher ANS fluorescence in pH 7 compared to 40 µM samples after initial decline. In both Figures 5a and 5b, the initial high ANS fluorescence in 120 µM samples may arise from HEWL aggregate opening up after suddenly experiencing electrostatic repulsions in pH 7. Subsequent drop in ANS fluorescence could arise when the protein has undergone conformational changes to minimize repulsions that may have sealed off some previously available binding sites, causing the population of ANS binding sites to decrease and stabilize. In Figure 5b, it is plausible that in 120 µM samples, a longer incubation in pH 12.2 fortifies the HEWL aggregate (with more disulphide bonds) against structural changes on exposure to pH 7. Thus non-polar pockets are significantly retained at pH 7 in these samples, while the same samples under shorter incubation periods in pH 12.2 probably undergoes conformational changes that perhaps internalize the exposed potential ANS binding sites and minimize bound ANS population to levels similar to 40 µM samples. Given that average integrated ANS fluorescence in pure buffer is ∼1.2×10^8^, it is apparent that ANS binding sites are few in 40 µM HEWL in comparison to 120 µM samples after 264 hours in pH 12.2. Thus, polycationic HEWL aggregates appear stable for a week in pH 7 when transferred after 134 hrs in pH 12.2, while exhibiting a significant intake capacity for non-polar molecules like ANS only when transferred from 120 µM samples.

## Discussion

### Model for HEWL self-assembly

**Figure 6:**
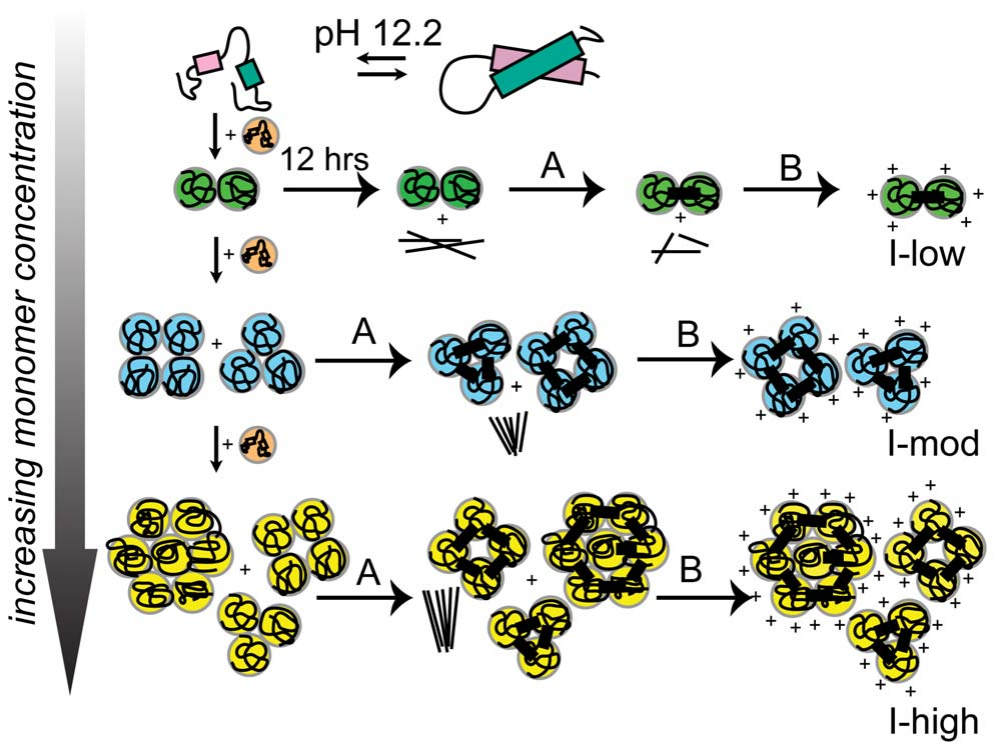
Schematic model for isodesmic aggregation of HEWL at pH 12.2. Exposure to pH(∼0.3 µM), moderate (3—10 µM) and high (120 µM) equilibrium aggregation intermediates or protein nanoparticles of increasing average size were obtained. *Step A* highlights formation of intermolecular disulphide bonds (thick bars) after 100 hours in pH 12.2. *Step B* yields stable polycationic HEWL nanostructures after dilution into 100 mM pH 7 sodium phosphate buffer after 134 hours in pH 12.2. Thin line clusters indicate amyloid fibrils formed on pathway.

**Figure 6** displays a schematic model to explain the observations. Exposure to pH 12.2 triggers rapid partial unfolding in native HEWL monomer, causing hydrophobic and backbone amide/carbonyl groups to become solvent exposed. Molecular encounters among such monomers accelerate non-covalent interactions, driving a downhill polymerization process forward, forming small oligomers initially. With time, the monomer population reduces with two consequences. Firstly, the likelihood of monomer-monomer encounter becomes much less, secondly, encounter of remaining monomers with an oligomer becomes frequent. The latter favours the growth of bigger aggregates with each aggregated species more stable than the next smallest species. Such encounters occur more frequently at high [M], explaining why large aggregates are prevalent at high [M]. Presently it is not clear if small oligomers also interact with each other to form aggregates. When [M] is low or [M] is depleted owing to oligomer formation, encounters are rare, so small oligomers are kinetically trapped with their growth arrested (Figs. 1a, 2b), forming intermediates (I_low_). Thus, concentration of monomers with exposed hydrophobic surfaces dictates the eventual size of the aggregate intermediate. Aggregate growth ceases after 12 hours (Figures 1a, 2b, 2d) because population of available monomers is depleted. Intermediates formed with low [M], show higher FRET efficiency (Figure 1a) due to small size, retain exposed hydrophobic surfaces as confirmed by tryptophan quenching (Figure S1) and dansyl emission (Figure 1b). However, they are unable to shield bound ANS (Figures 1c–d), owing to absence of deep hydrophobic pockets unlike larger aggregate intermediates (I_high_) which are formed with higher [M]. Aggregates of multiple sizes at moderate and high concentrations in Figure 6 reflect the size heterogeneity in the aggregate ensemble. Such heterogeneity is consistent with broad distribution observed in DLS data (Figure 2e) and smear observed previously among the electrophoretic gel stains of HEWL aggregates [16], [28].

Aggregation of HEWL at pH 12.2 exhibits the following characteristics: a) an absence of critical concentration for aggregation to be initiated (Fig. 1a), b) nonexistence of a lag phase (Figs. 1a,2b) before aggregation probably because no conformational rearrangement is needed for aggregates to form, implying no rate determining step, c) aggregate growth kinetics governed by a monomer concentration independent rate constant (Table 1 **)** and d) presence of monomer in equilibrium with aggregates (Fig. 2e). The above features argue for an isodesmic polymerization mechanism. Indeed the observed variation in r_ss_ against time (Fig. 2b) is identical with profiles reported previously [32] for variation of the degree of polymerization for different [M] against time in an isodesmic model (see Fig. 11[32]). A monomer-dimer/isodesmic model has been proposed earlier for HEWL association at vastly higher HEWL concentrations(1.5—10 mM), acidic pH (3–8) in presence of 0–0.5 M NaCl [33].

The aggregates or nanoparticles are toughened by intermolecular disulphide bonds after 100 hours in pH 12.2 [16], which enables them to withstand strong coulombic repulsions in their polycationic form at pH 7. In the absence of disulphide bonds to fasten them, the 120 µM aggregates fall apart as observed when transferred to pH 7 at early times (Fig. 4a,c). The absence of intermolecular disulphide bonds at early times explains why HEWL samples in pH 12.2 recover enzymatic activity when transferred to pH 7 early on (Fig. 2a). Interestingly the loss in enzymatic activity is not dependent on size of aggregates and therefore not influenced by the restrictive growth mechanism. The slow formation of inappropriate disulphide bonds during long incubation in pH 12.2 probably curtails the protein chain dynamics that is critical for enzyme function resulting in loss of enzymatic activity. The intermolecular disulphide bonds therefore serve an important role of fastening the monomers together in the aggregate, not withstanding loss in HEWL enzymatic activity.

Intermolecular disulphide bonds in HEWL have been shown to promote the growth of large aggregates by strengthening the non-covalent forces [34]. Earlier work from our lab had revealed that presence of DTT in the aggregation medium strongly inhibits formation of HEWL aggregates, highlighting the critical role of disulphide bonds in HEWL aggregation at alkaline pH [16]. The role of incorrect disulphide bonds in promoting aggregation is a symptom of several diseases like amyotrophic lateral sclerosis and cataract. Thus this makes HEWL aggregation as a good model system to investigate the etiology of such diseases.

Polycationic HEWL aggregates appear competent to bind non-polar molecules like ANS (Fig. 4c,d and Fig. 5) due to availability of hydrophobic space perhaps owing to flexible molecular packing in the interiors. Aggregates from 120 µM samples bind more ANS molecules (Fig. 5b) consistent with their larger size in comparison aggregates from 40 µM samples. At this point it is not clear if ANS is encapsulated inside the larger aggregates from 120 µM samples. Such binding can have potential applications of polycationic HEWL aggregates as a drug nanocarrier or DNA/RNA carrying protein nanoparticle.

Two novel features are noteworthy among HEWL amyloid fibrils. First, the fibrils appear in 300 nM HEWL samples soon after 12 hours in pH 12.2. Second multiple fibrils are seen growing outward from amorphous aggregates in AFM images (Fig. 3C,G,H). The latter is a direct confirmation of on-pathway formation of amyloid fibrils from amorphous aggregates. Previous reports on aggregation and amyloid formation of HEWL at acidic pH stipulate incubation of high monomer concentrations (>1 mM) for several weeks at 37—57°C [12], [35]. Presumably, the electrostatic repulsions between large positively charged HEWL molecules act as a barrier against amyloid formation. In contrast, the net charge of HEWL at pH 12.2 is likely to be small owing to its pI at 11.3, thus enabling facile formation of oligomers and amyloid fibrils even at sub-micromolar concentrations. Indeed the experimentally measured and theoretically simulated values second virial coefficient (B_2_) of lysozyme has been plotted against pH [36]. They both reveal a clear decrease in B_2_ (positive to negative) with increasing pH (4.5 to 10.5). This decrease is shown to extend till isoelectric pH (∼11.3) in another report [37]. Our observations at pH 12.2 suggest that B_2_ continues to remain negative at pH 12.2. Thus van der Waals attractions begin to dominate over electrostatic repulsions in HEWL as pH becomes more alkaline.

We observed that at short incubation times (∼12 hours), fibrils were more frequently seen at lower monomer concentrations (≤ 3 µM) compared to higher (≥ 50 µM) using AFM. Indeed it has been predicted that in the limit of high monomer concentrations, rate of molecular contact between a growing fibril and a monomer, varies inversely with protein concentration. Further monomer addition to fibril is said to progress by dock-and-lock mechanism, such that the rate-determining step in the monomer addition is the lock phase in which both the preformed oligomer and the monomer undergo combined conformational changes that form a stable antiparallel higher-order oligomer [38]. In view of their small size and unhindered dynamics, oligomers at low concentrations probably adapt their conformation more rapidly to enable locking onto the fibril. Perhaps, lower concentrations allow sufficient relaxation time for oligomers to attain right orientation to lock into ordered aggregates**,** like in protein crystallization [39].

*In silico* approaches to model the aggregation of proteins are now emerging [40]. Such methods can yield residue wise structural information on the different aggregate species populated at different time points during aggregation. For example, it has been shown that dimer formation in Aβ peptide can lock few misfolded conformations and shift equilibrium away from native state [41].

The self-association of HEWL in aqueous medium has been modeled theoretically previously. Carlsson et. al., have investigated the self-association of HEWL (modeled as a hard sphere) by Monte Carlo simulations as a function of protein concentration, pH and ionic strength of surrounding medium [37]. Their results reveal that HEWL aggregation is promoted by increasing monomer concentration and conditions causing decreased electrostatic repulsion between protein monomers. The latter can be achieved by either increasing pH (which diminishes net charge) or increasing ionic strength (which screens out electrostatic repulsions). Our results in this work reflect a similar trend. At pH 12.2 employed in our study, the low net charge facilitates HEWL aggregation at 300 nM which is about ∼1000 fold lower than the lowest concentration employed by Carlsson and coworkers. The increase in average size of aggregates with increasing protein concentration (Figures 2b, 2c, 2d) observed by us is consistent with the postulated role of increasing protein concentration in promoting aggregation.

Pellicane has modeled protein interactions in HEWL aqueous solution using the Derjaguin-Landau-Verwey-Overbeek (DLVO) theory [42]. With increase in pH or ionic strength it was predicted that short range attraction competes with long range repulsion to promote formation of aggregates. In our case, at pH 12.2, repulsions are likely to be less owing to diminished net charge making aggregation spontaneous as observed by us and predicted by DLVO theory. Indeed it has been hypothesized that protein aggregation is caused by maximizing van der Waals interaction between side chains and backbone hydrogen bonds [41]. In our study of HEWL aggregation at alkaline pH, the formation of intermolecular disulphide bonds is a *unique* feature that is not accounted by any theory or model so far. In another work [34], it has been demonstrated that presence of such disulphide bonds reinforces the otherwise weak non-covalent forces that hold the aggregates together. This probably explains the robust nature HEWL aggregates in our study.

Earlier work with HEWL under acidic condition (pH 1.6 and 65°C) has revealed no amyloid fibril formation with 50 µM lysozyme upto 170 hours, suggesting a critical concentration >50 µM at this condition[43]. It was also shown that fragmentation of the HEWL polypeptide by acid hydrolysis forming the 49–101 peptide was essential for efficient fibril formation. In contrast, our earlier work [16] has shown that significant fragmentation of HEWL at pH 12.2 occurs only after 16 days, hence it is unlikely that fragmentation has any role in fibril formation at alkaline pH after 12 hours. In another work under acidic conditions (1.2 mM HEWL, pH 2.0 with 175 mM NaCl), HEWL monomers have been shown to form uniformly sized oligomers (R_h_ ∼3.8 nm) which after reaching a threshold concentration triggered protofibril nucleation. Later protofibrils grew when oligomers added on to ends of protofibrils [44].It is likely that high salt is essential for screening out large net positive charge in HEWL at acidic pH to facilitate protein-protein interaction, unlike our case here. Moreover, no curly worm-like protofibrils were observed by us in AFM suggesting perhaps their transient nature. Thus it is evident that rapid amyloid formation by HEWL at sub-micromolar concentrations is exclusively observed at pH 12.2.

## Conclusions

We have isolated and characterized multiple oligomeric intermediates on the aggregation pathway of HEWL in pH 12.2 at room temperature. Scrutiny of the structure, function and dynamics of these intermediates revealed a stepwise incremental increase in average size, internal molecular packing coupled with decrease in enzymatic activity on maturation of aggregates suggesting an isodesmic polymerization mechanism. Direct observation of amyloid fibrils growing from amorphous HEWL aggregates in AFM images, confirmed the on-pathway formation of fibrils. Further, owing to unique formation of intermolecular disulphide bonds these aggregates remain stable in their polycationic form at neutral pH akin to protein nanoparticles. Such stability augurs well for high resolution structural studies on these isolated intermediates that can shed light on mechanism of the on-pathway formation of amyloid fibrils from oligomers. Our results suggest that amyloid fibril formation in HEWL at pH 12.2 is quick and facile at sub-micromolar concentrations unlike other conditions. Finally the isolated intermediates can be tried and tested as prospective nanocarriers for non-polar drugs or polyanionic nucleic acids.

## Supporting Information

Figure S1 **Fluorescence quenching of tryptophan(s) in HEWL by iodide during aggregation. A.** Quenching of tryptophan steady state fluorescence in HEWL (0.3 µM, 3 µM and 120 µM) and model compound N-acetyl-L-tryptophanamide (NATA, 5 µM) are shown at various time intervals after incubation at pH 12.2. **B.** *Fitted Stern-Volmer plots for fluorescence quenching of tryptophan by iodide during aggregation*. Stern-Volmer plots for quenching of tryptophan in 0.3 µM HEWL are shown in top row for different incubation times in pH 12.2 starting from 0 h (leftmost) followed by 6, 12 and 24 hrs (rightmost). Similar plots are shown for 3 µM HEWL (second row from top), 120 µM HEWL (third row) and 3 µM NATA (bottom row). The dashed lines reveal the linear regression fit used to calculate the K_SV_.
(TIF)

Figure S2 **Mean fluorescence lifetime (τ_m_) of dansyl-HEWL conjugates during aggregation.** The variation in mean fluorescence lifetime of dansyl probe in dansyl-HEWL conjugates is shown against time of incubation in pH 12.2 for different HEWL concentrations. The filled symbols show the mean lifetime (τ_m_), measured from emission collected at 438 nm using a monochromator, while unfilled symbols show mean lifetime measured from whole emission collected using an excitation cutoff filter.
(TIF)

Figure S3 **Molecular mass and size of HEWL nanoparticles determined by MALS.** The molecular mass and hydrodynamic radius of 120 µM HEWL samples, after 7 days of incubation in pH 12.2 at room temperature, as determined using multi-angle light scattering is shown. Note the minor presence of a low molecular weight fraction similar to Fig. 2e.
(TIF)

Figure S4 **Morphology of HEWL amyloid fibrils.** Observed morphology of HEWL fibrils using atomic force microscopy with different monomer concentrations incubated as indicated is shown. The height of the fibril may be estimated from observed range in Z axis indicated in square brackets with minimum value being darkest region in image and maximum value being most white. Details of each image are as follows: **A**: 3 µM, pH 12.2, 12 hours, [0—1.5 nm]; **B**: 50 µM, pH 12.2, 25 days, [0—0.8 nm]; **C**: 160 µM, pH 12.2, 31 days, [0—1.15 nm] and **D**: 75 µM, pH 12.2, 30 days, [0—2.0 nm]. Scale bar  =  100 nm.
(TIF)

Figure S5 **Concentration of free –SH in HEWL during aggregation using DTNP (2,2’-dithiobis(5-nitropyridine)).** The variation of free –SH in HEWL (diluted to 2 µM in assay cuvette) incubated at concentrations 20, 50 & 120 µM in pH 12.2 is shown against time. HEWL has eight Cys residues per polypeptide. The assay procedure used has been described previously (16).
(TIF)

Figure S6 **Emission maxima of ANS in presence of HEWL nanoparticles transferred to pH 7.** The variation in emission maxima of ANS in presence of HEWL nanoparticles with time after 10-fold dilution to pH 7 from 120 (Fig. 4c) or 40 (Fig. 4d) µM samples.
(PDF)

Table S1 **Parameters extracted (using eq. 5) from tail fit analysis of fluorescence anisotropy decays shown in** **Figure 2c** **.** (Note: χ^2^ reported here is reduced chisquare).
(PDF)

Table S2 **Observed average ± std. dev. of global rotational correlation time (φ_2_) extracted from multiple experiments after 12 and 24 hrs of aggregation displayed in** **Figure 2d** **.**
(PDF)
