# Supplementary figures and images for: On the Characterization of Intermediates in the Isodesmic Aggregation Pathway of Hen Lysozyme at Alkaline pH
Source: PLoS One. 2014 Jan 28;9(1):e87256. doi: 10.1371/journal.pone.0087256 (PMC3904990; doi:10.1371/journal.pone.0087256)

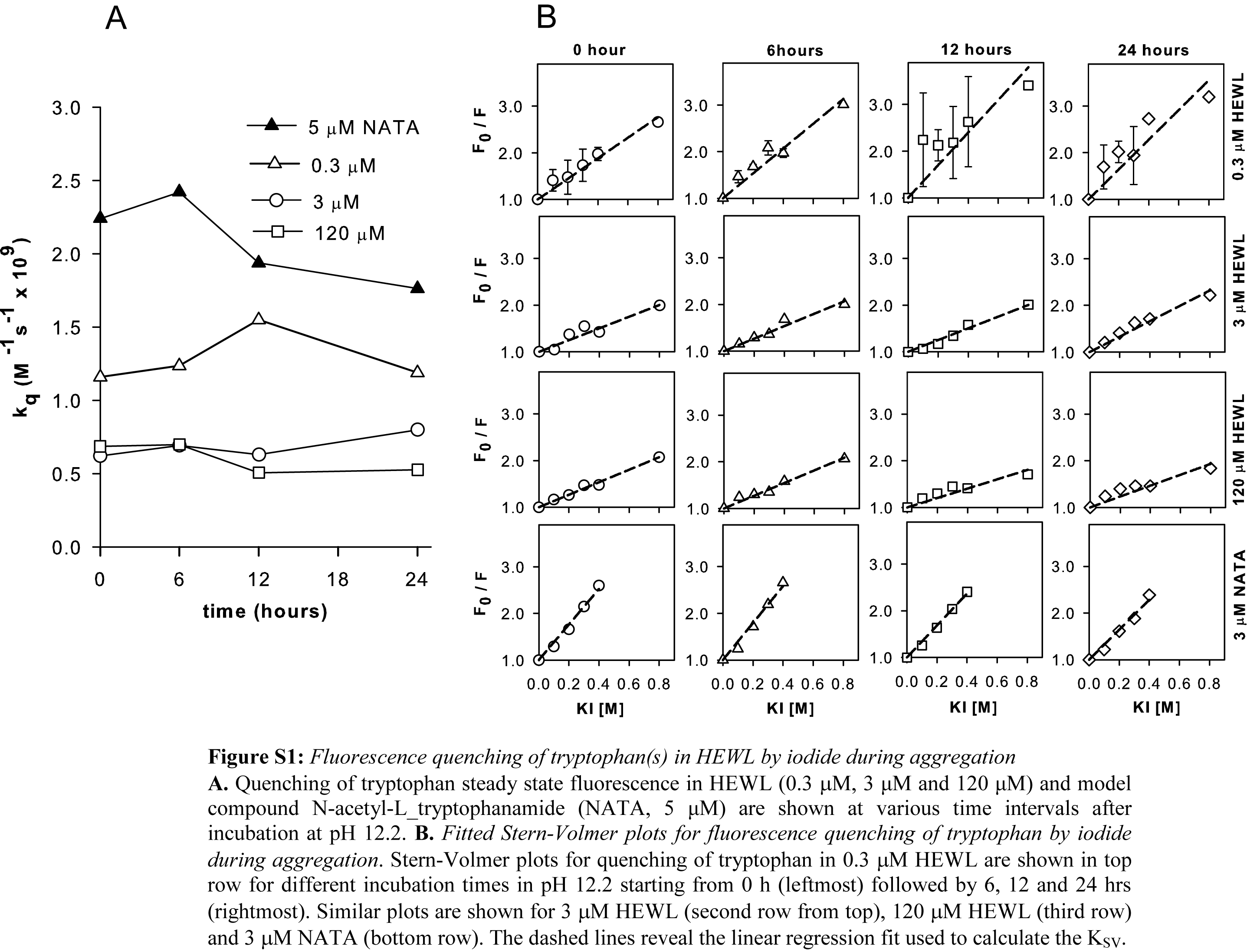

Supplement: Figure S1 — Fluorescence quenching of tryptophan(s) in HEWL by iodide during aggregation. A. Quenching of tryptophan steady state fluorescence in HEWL (0.3 µM, 3 µM and 120 µM) and model compound N-acetyl-L-tryptophanamide (NATA, 5 µM) are shown at various time intervals after incubation at pH 12.2. B. Fitted Stern-Volmer plots for fluorescence quenching of tryptophan by iodide during aggregation. Stern-Volmer plots for quenching of tryptophan in 0.3 µM HEWL are shown in top row for different incubation times in pH 12.2 starting from 0 h (leftmost) followed by 6, 12 and 24 hrs (rightmost). Similar plots are shown for 3 µM HEWL (second row from top), 120 µM HEWL (third row) and 3 µM NATA (bottom row). The dashed lines reveal the linear regression fit used to calculate the KSV. (TIF) [file pone.0087256.s001.tif]

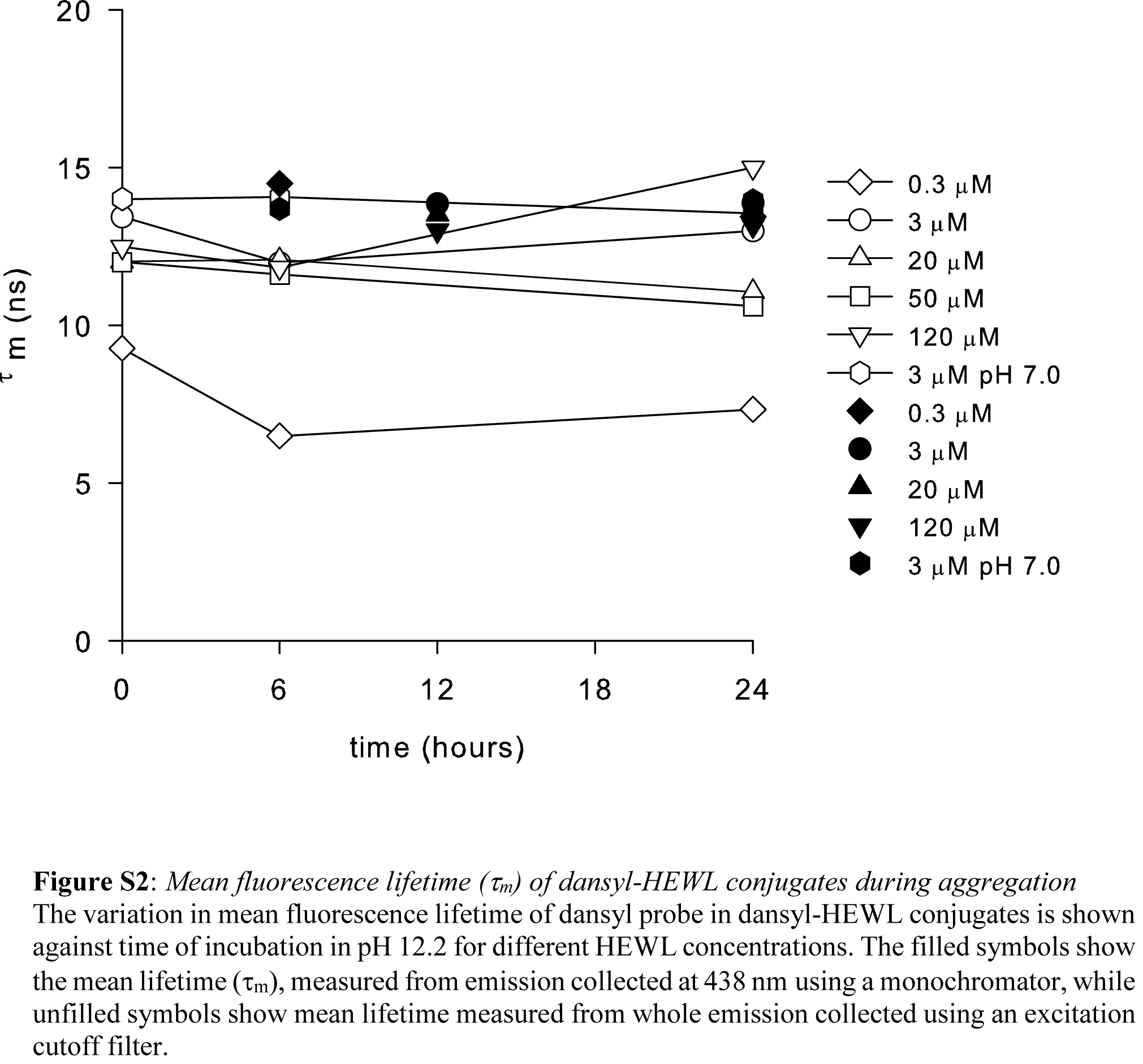

Supplement: Figure S2 — Mean fluorescence lifetime (τm) of dansyl-HEWL conjugates during aggregation. The variation in mean fluorescence lifetime of dansyl probe in dansyl-HEWL conjugates is shown against time of incubation in pH 12.2 for different HEWL concentrations. The filled symbols show the mean lifetime (τm), measured from emission collected at 438 nm using a monochromator, while unfilled symbols show mean lifetime measured from whole emission collected using an excitation cutoff filter. (TIF) [file pone.0087256.s002.tif]

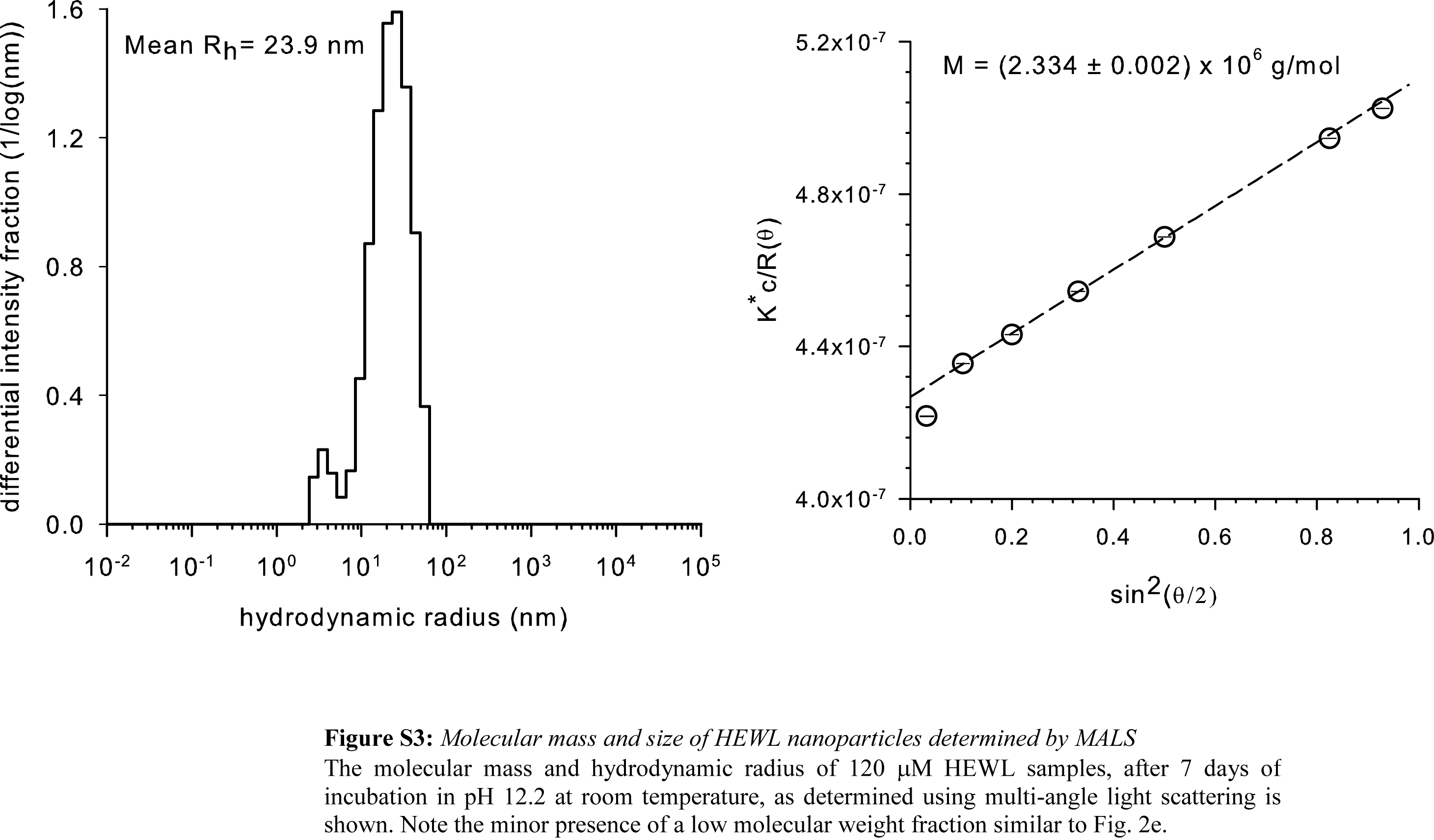

Supplement: Figure S3 — Molecular mass and size of HEWL nanoparticles determined by MALS. The molecular mass and hydrodynamic radius of 120 µM HEWL samples, after 7 days of incubation in pH 12.2 at room temperature, as determined using multi-angle light scattering is shown. Note the minor presence of a low molecular weight fraction similar to Fig. 2e. (TIF) [file pone.0087256.s003.tif]

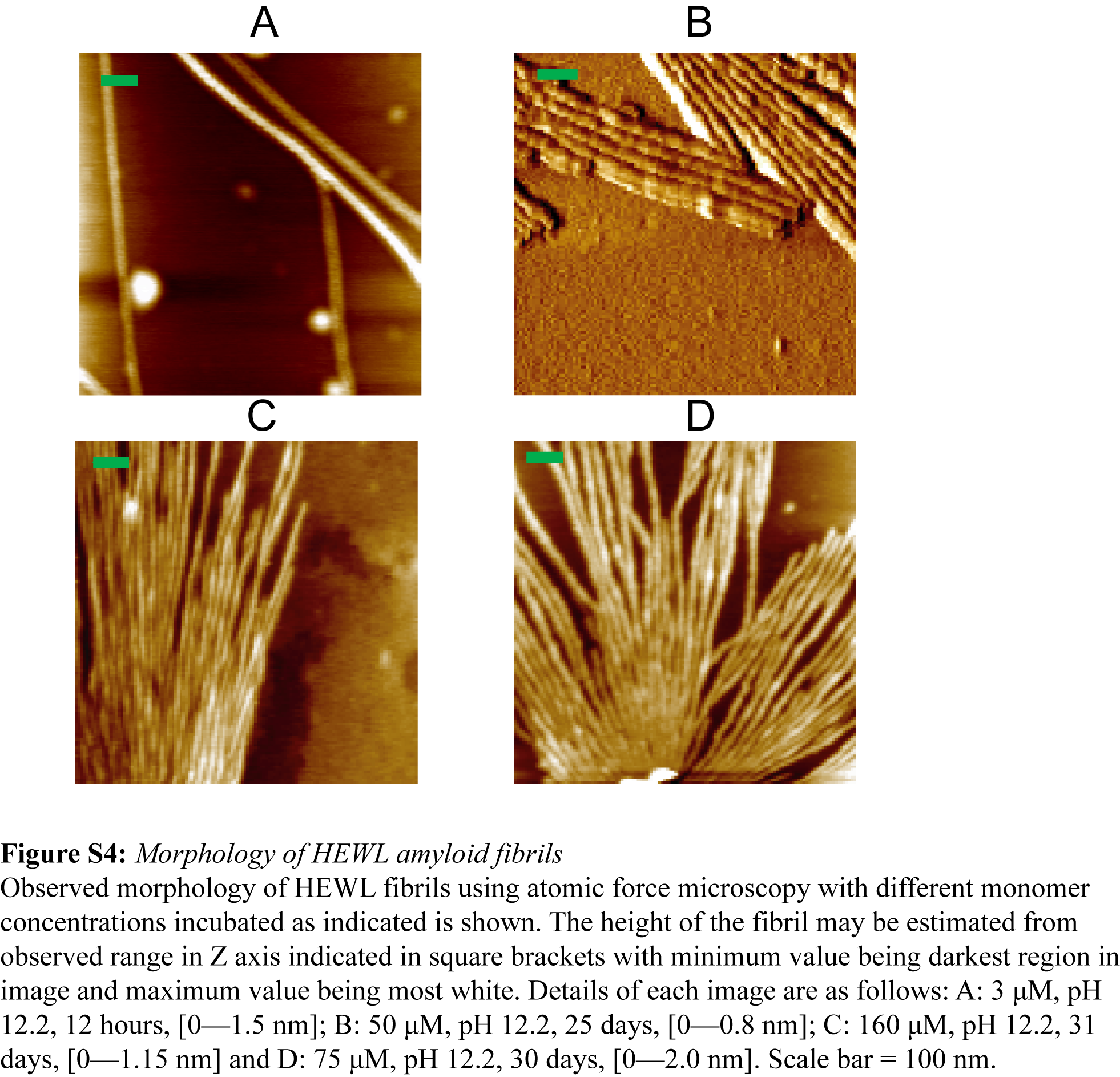

Supplement: Figure S4 — Morphology of HEWL amyloid fibrils. Observed morphology of HEWL fibrils using atomic force microscopy with different monomer concentrations incubated as indicated is shown. The height of the fibril may be estimated from observed range in Z axis indicated in square brackets with minimum value being darkest region in image and maximum value being most white. Details of each image are as follows: A: 3 µM, pH 12.2, 12 hours, [0—1.5 nm]; B: 50 µM, pH 12.2, 25 days, [0—0.8 nm]; C: 160 µM, pH 12.2, 31 days, [0—1.15 nm] and D: 75 µM, pH 12.2, 30 days, [0—2.0 nm]. Scale bar = 100 nm. (TIF) [file pone.0087256.s004.tif]

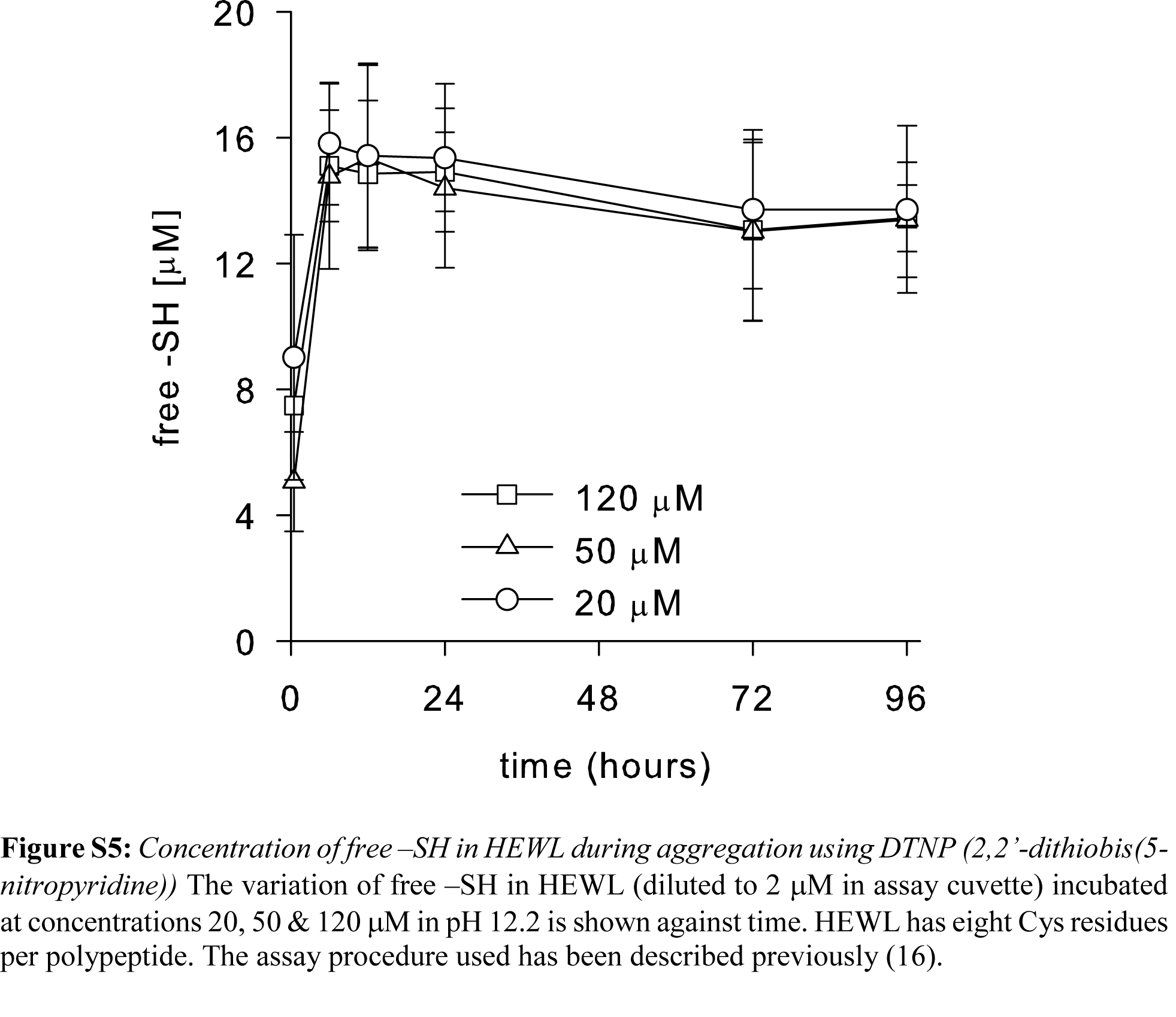

Supplement: Figure S5 — Concentration of free –SH in HEWL during aggregation using DTNP (2,2’-dithiobis(5-nitropyridine)). The variation of free –SH in HEWL (diluted to 2 µM in assay cuvette) incubated at concentrations 20, 50 & 120 µM in pH 12.2 is shown against time. HEWL has eight Cys residues per polypeptide. The assay procedure used has been described previously (16). (TIF) [file pone.0087256.s005.tif]
