# Supplementary material for: On the Characterization of Intermediates in the Isodesmic Aggregation Pathway of Hen Lysozyme at Alkaline pH
Source: PLoS One. 2014 Jan 28;9(1):e87256. doi: 10.1371/journal.pone.0087256 (PMC3904990; doi:10.1371/journal.pone.0087256)
Supplement: Figure S6 — Emission maxima of ANS in presence of HEWL nanoparticles transferred to pH 7. The variation in emission maxima of ANS in presence of HEWL nanoparticles with time after 10-fold dilution to pH 7 from 120 (Fig. 4c) or 40 (Fig. 4d) µM samples. (PDF) [file pone.0087256.s006.pdf]

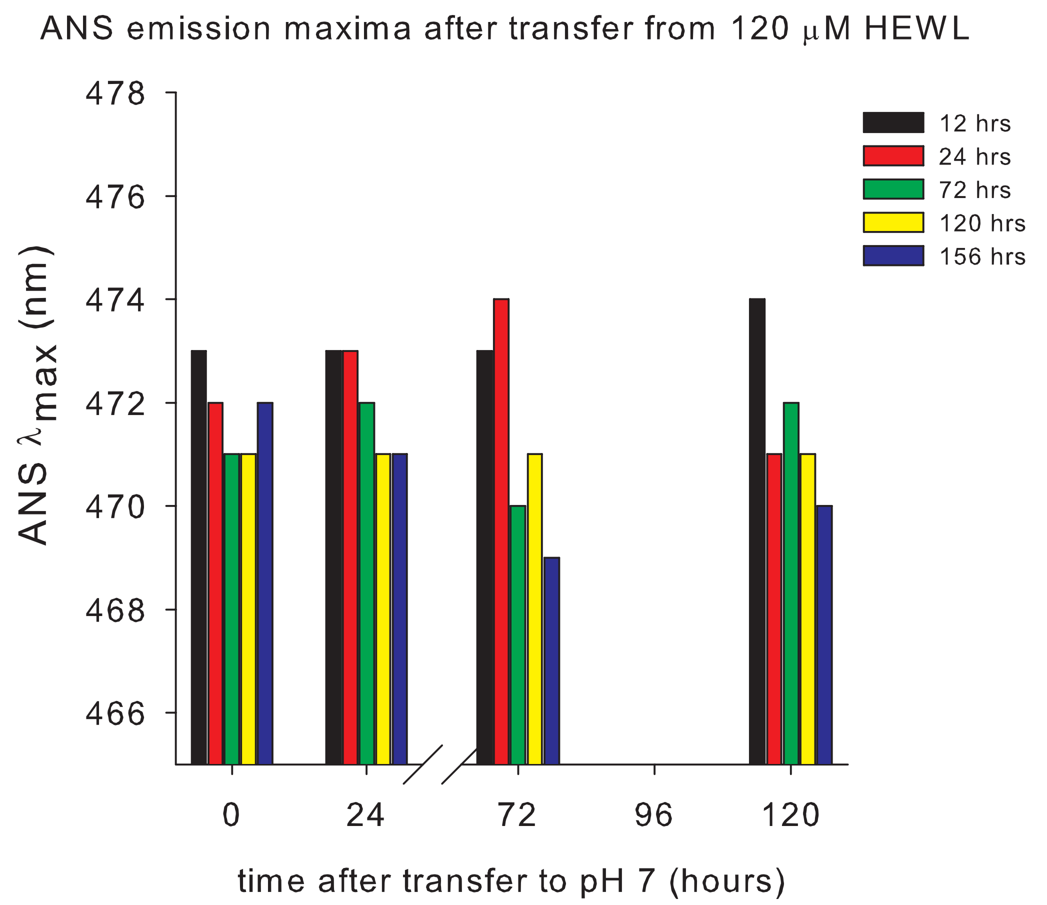

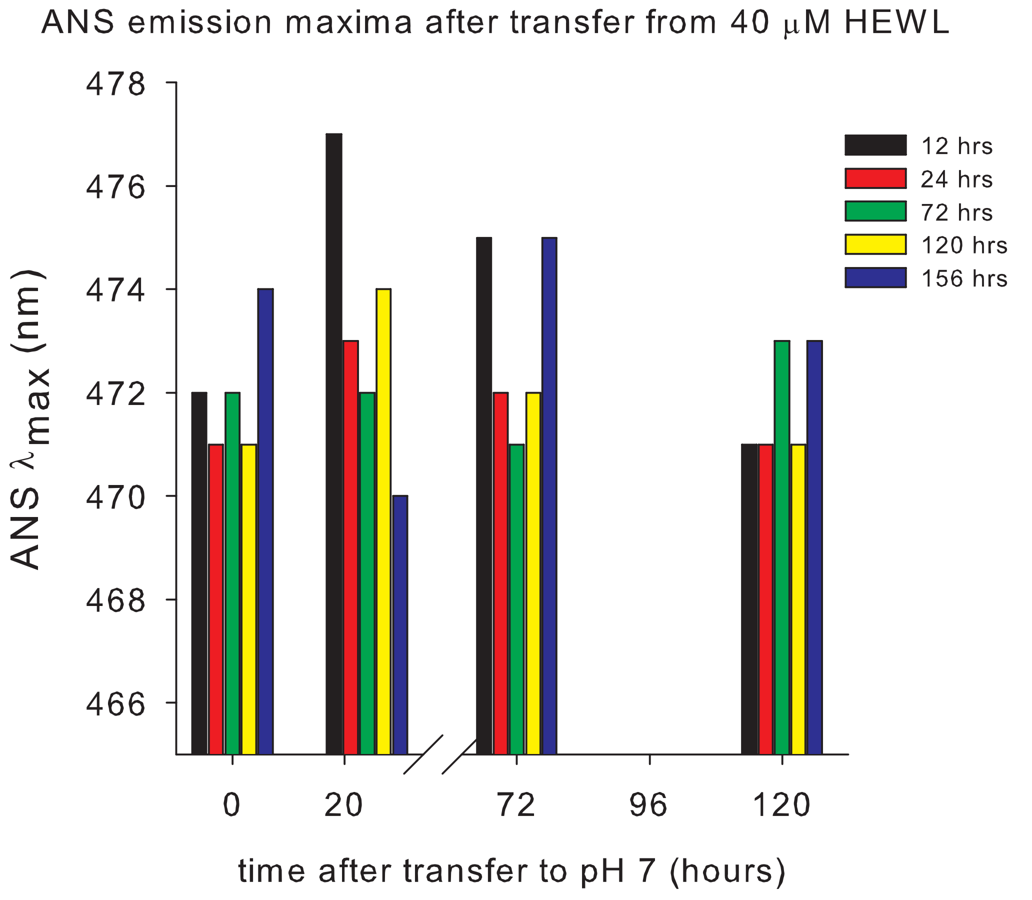

Figure S6: *Emission maxima of ANS in presence of HEWL nanoparticles transferred to pH 7*
The variation in emission maxima of ANS in presence of HEWL nanoparticles with time after 10-fold dilution to pH 7 from 120 (Fig. 4c) or 40 (Fig. 4d) μM samples.
