# Supplementary material for: On the Characterization of Intermediates in the Isodesmic Aggregation Pathway of Hen Lysozyme at Alkaline pH
Source: PLoS One. 2014 Jan 28;9(1):e87256. doi: 10.1371/journal.pone.0087256 (PMC3904990; doi:10.1371/journal.pone.0087256)
Supplement: Table S1 — Parameters extracted (using eq. 5) from tail fit analysis of fluorescence anisotropy decays shown in Figure 2c . (Note: χ2 reported here is reduced chisquare). (PDF) [file pone.0087256.s007.pdf]

Table S1: Parameters extracted (using eq. 5) from tail fit analysis of fluorescence anisotropy decays shown in Figure 2c. (Note: χ^2 reported here is reduced chisquare)

Figure	[HEWL], pH	Time of incubation (hours)	A	ϕ_1 (ns)	ϕ_2 (ns)	β_1	β_2	χ^2
2c A	3 µM, pH 7.0	24	0.000	0.14	**4.3**	0.08	0.92	1.1
2c B	0.3 µM, pH 12.2	24	0.00	0.12	**5.3**	0.09	0.91	1.01
2c C	3 µM, pH 12.2	24	0.004	0.24	**9.8**	0.03	0.97	1.02
2c D	20 µM, pH 12.2	24	0.002	0.54	**15**	0.02	0.98	1.03
2c E	120 µM, pH 12.2	24	0.002	0.76	**26**	0.01	0.99	0.99
