# Supplementary material for: On the Characterization of Intermediates in the Isodesmic Aggregation Pathway of Hen Lysozyme at Alkaline pH
Source: PLoS One. 2014 Jan 28;9(1):e87256. doi: 10.1371/journal.pone.0087256 (PMC3904990; doi:10.1371/journal.pone.0087256)
Supplement: Table S2 — Observed average ± std. dev. of global rotational correlation time (φ2) extracted from multiple experiments after 12 and 24 hrs of aggregation displayed in Figure 2d . (PDF) [file pone.0087256.s008.pdf]

Table S2: Observed average ± std. dev. of global rotational correlation time (ϕ_2) extracted from multiple experiments after 12 and 24 hrs of aggregation displayed in Figure 2d.

[HEWL], pH	Time of incubation (hours)	ϕ_2 (ns)
3 µM, pH 7.0	12	4.5 ± 0.01
0.3 µM, pH 12.2	12	7.9 ± 0.8
3 µM, pH 12.2	12	10.6 ± 0.4
20 µM, pH 12.2	12	14.5 ± 2.4
120 µM, pH 12.2	12	26.9 ± 2.3
3 µM, pH 7.0	24	4.4 ± 0.04
0.3 µM, pH 12.2	24	6.9 ± 2.2
3 µM, pH 12.2	24	9.7 ± 0.2
20 µM, pH 12.2	24	14.7 ± 0.3
120 µM, pH 12.2	24	25.8 ± 0.8
